# Antimicrobial Nonwoven Fabrics Incorporated with Levulinic Acid and Sodium Dodecyl Sulfate for Use in the Food Industry

**DOI:** 10.3390/foods11152369

**Published:** 2022-08-07

**Authors:** Zijun Liu, Haiqi Long, Yihan Wang, Cangliang Shen, Dong Chen

**Affiliations:** 1 College of Food Science, Southwest University, 2 Tiansheng Rd, Beibei, Chongqing 400715, China; 2 National Demonstration Center for Experimental Food Science and Technology Education, Southwest University, Chongqing 400715, China; 3 Chongqing Key Laboratory of Speciality Food Co-Built by Sichuan and Chongqing, Chongqing 400715, China; 4 Department of Food Quality and Safety, Westa College, Southwest University, 2 Tiansheng Rd, Beibei, Chongqing 400715, China; 5 Division of Animal and Nutritional Sciences, West Virginia University, Morgantown, WV 26506, USA

**Keywords:** aerosol, antivirus, face mask, filter, levulinic acid, SDS

## Abstract

Safe and cost-effective antimicrobial fabrics (e.g., face masks and air filters) are conducive to preventing the spread and transmission of respiratory microorganisms in food processing plants and retail establishments. The objective of this study was to coat fabrics with two commonly used compounds in the food industry: levulinic acid (LVA) and sodium dodecyl sulfate (SDS) and determine the antimicrobial efficacy of the coated fabrics against bacterial solutions, aerosols, and influenza A virus subtype H1N1. In addition, air permeability and shelf-life of the LVA/SDS coated fabrics were also examined. Nonwoven fabrics were dip-coated with three concentrations (*w*/*v*, 0.5% LVA + 0.1% SDS, 1% LVA + 0.5% SDS, and 2% LVA + 1% SDS) of LVA and SDS and challenged with bacterial solutions (*Staphylococcus aureus* and *Escherichia coli*, ca. 7.0 log CFU/coupon) for a contact time of 3, 5, and 10 min. The coated fabrics were also challenged with *S. aureus* aerosol and H1N1 virus following standard operations of ASTM F2101-19 and ISO 18184:2019, respectively. The 1% LVA + 0.5% SDS coated fabrics showed potent antibacterial efficacy against both bacterial solutions (>6.0-log reduction to under the detection limit of 1.0 log CFU/coupon for *S. aureus*; ca. 1.0-log reduction for *E. coli*) and aerosols (>3.6-log reduction to under the detection limit), with greater inactivation occurring at higher concentrations and longer exposure time. Moreover, the coated fabrics inactivated >99% of the H1N1 virus. The shelf-life of the coated fabrics was stable within 12 months and the air permeability was not adversely affected with the coating concentrations less than 1% LVA + 0.5% SDS. Results reveal these low-cost and safe materials have the potential to be used to coat fabrics in the food industry to combat the spread and transmission of pathogens.

## 1. Introduction

Face masks and air filters are widely used in food processing plants and retail establishments to prevent the transmission of pathogenic bioaerosol [1,2]. The face mask is a piece of protective equipment routinely worn by employees in the food production environment. During the current COVID-19 pandemic, it plays a more significant role in the food processing surroundings since severe acute respiratory syndrome coronavirus 2 (SARS-CoV-2) can easily infect humans in poorly ventilated indoor spaces, and a face mask is the most effective prevention against the spreading of respiratory droplets and aerosols [2]. Although so far there has been no epidemiological evidence for foodborne transmission of SARS-CoV-2, the theoretical risk of its transmission via foods cannot be ruled out since a traditional epidemiological foodborne investigation is unlikely to be carried out on the COVID-19 infected patients [3]. The virus could be introduced via cold chain transportation of contaminated foods (e.g., salmon), and might initiate an outbreak [4]. Other respiratory viruses, including avian influenza viruses, parainfluenza viruses, orthopneumovirus, rhinoviruses, and Nipah virus, are all concerns in the food industry, causing diseases either in humans and/or farm animals and causing significant economic losses [3].

Air filters are other fabrics widely used in the ventilation systems of enclosed hatcheries, farms, and slaughterhouses to prevent the introduction and transmission of airborne bacteria and viruses [1]. However, the most commonly used face masks and filters are made of fabrics lacking antimicrobial properties against either viruses or bacteria. Considering the food processing surroundings (e.g., communication by shouting in noise, limited ventilation, close spacing for long periods, etc.) and how labor-intensive the industry is, viable microbial loads escaping from the masks worn by infected food handlers, either symptomatic or asymptomatic, could infect others [5]. Moreover, microbial transmission can take place by touching the mask, and discarded masks are a source of contaminated biological waste posing a potential threat to the handlers [6]. Similarly, currently used filters can block certain viruses from entering facilities but cannot inactive the viral agents. Airborne microorganisms that accumulate on the surface of the filters may survive for weeks or even months, and then contaminate the farm animals and workers if they are not handled and disposed of properly [7,8].

Fabrics with antimicrobial functions have been widely researched recently, but few of these are really applicable in the food processing environments due to the cost-ineffective materials (e.g., graphene, copper, silver, etc.) [9,10,11,12,13], high-risk chemicals (e.g., formaldehyde) used in the manufacturing [14], and/or instability of antimicrobial effectiveness at practical settings [15]. Therefore, we report an easy-to-apply method for developing antimicrobial fabrics by incorporating two commonly used compounds in the food industry: levulinic acid (LVA) and sodium dodecyl sulfate (SDS). LVA is a 5-carbon organic acid that has been designated as Generally Recognized as Safe (GRAS, 21 CFR, 172.515) by the U.S. Food and Drug Administration (FDA) for direct addition to foods as a flavoring agent or adjunct [16]. SDS, an anionic surfactant, also has GRAS status (21 CFR 172.822) for being used as a multipurpose additive in a variety of foods, including egg whites, fruit juices, gelatin, and vegetable oils as a whipping or wetting agent [17]. Hence, the concern for their safety is likely minimal. The main ingredient, LVA, can be produced at a comparatively low cost (approximately 20 cents/L for 3% LVA, *w*/*v*) yet in a high yield from renewable feedstocks [18]. The use of LVA and SDS alone has limited antimicrobial efficacy on tested microorganisms, and synergism between them has been observed [19]. Our previous study indicated that a combination of 3% LVA and 2% SDS reduced >6.9 log CFU/coupon of foodborne pathogens in biofilms formed on stainless steel within 10 min [20]. Co-application of 5% LVA and 2% SDS inactivated murine norovirus by 2.5 1og PFU/mL in an aqueous suspension, and by >1.50 log PFU/mL on inoculated stainless steel, after a 1-min exposure, and the numbers were reduced to undetectable levels with 5 min of application by 0.5% LVA plus SDS [21]. These studies indicate that the combination of LVA and SDS has a potent antibacterial and antiviral efficacy, showing the potential to be applied in the modification of fabrics widely used in the food industry.

In this study, melt-blown nonwoven fabrics were incorporated with LVA and SDS, and the coated fabrics were challenged with both Gram-positive and Gram-negative bacteria, bioaerosols, and viruses. In addition, verification of coating, air permeability, and shelf-life of the coated fabrics were also determined to evaluate the feasibility of LVA/SDS coated fabrics.

## 2. Materials and Methods

### 2.1. Experimental Design

**Figure 1:**
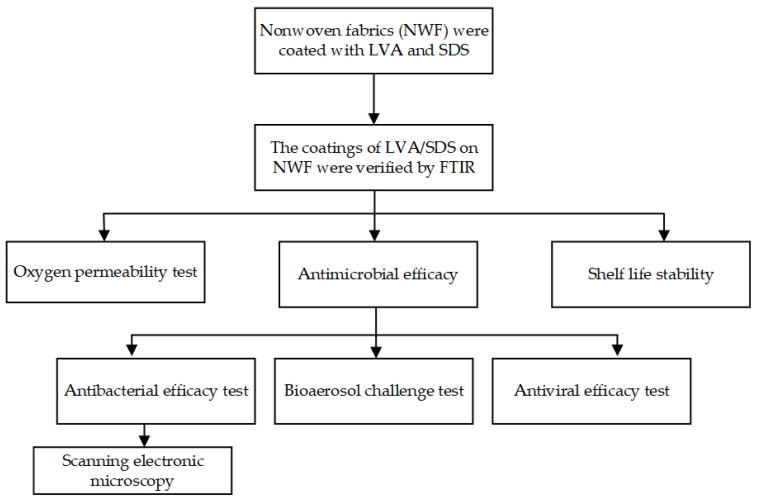
Procedure of the experiment.

In order to coat nonwoven fabrics (NWF) with LVA plus SDS and determine the feasibility of the NWF + LVA/SDS with antimicrobial capabilities to be used in the food industry, two sets of experiments were performed. The first set of experiments addressed the coating procedure, verification of the coating, oxygen permeability, and shelf-life stability. Three concentrations of LVA plus SDS were coated on NWF coupons by a dip-coating method, and the coating was verified by Fourier transform infrared spectroscopy (FT-IR). An oxygen transmission rate test was carried out at 65% relative humidity and 21 °C to mimic the surroundings that the antimicrobial fabrics may encounter during the food processing conditions. Shelf-life stability was tested by the antibacterial effectiveness stored for up to 12 months under an ambient environment. The second set of experiments focused on the performance of antimicrobial functions of the coated fabrics in a worst-case scenario. The sandwich test and direct plating method were applied to determine the antibacterial efficacy of the samples. A bioaerosol of *S. aureus* was dispensed by an apparatus assembled in the lab, and the coated coupons were continuously challenged for 6 h. Antiviral efficacy test was performed using the TCID_50_ method. The detailed experimental design is shown in Figure 1.

### 2.2. Coating Procedure

Nonwoven fabrics (50 g/m^2^) were taken from the U.S. National Institute for Occupational Safety and Health (NIOSH) certified N95 respirators (1860, 3M, St. Paul, MN, USA), except otherwise specified, and aseptically cut into square coupons (ca. 1 × 1 cm). The coupons were then soaked for 90 s in 500 mL of 75% ethanol containing three different concentrations (*w*/*v*, 0.5% LVA + 0.1% SDS, 1% LVA + 0.5% SDS, and 2% LVA + 1% SDS) of LVA (Macklin, Shanghai, China) and SDS (Solarbio, Beijing, China) with a stirring rate of 500 rpm. Coupons treated with 75% ethanol only were the control. All the coupons were then placed vertically leaning against the edge of Petri dish bases and air dried at room temperature for 24 h before use.

### 2.3. Fourier Transform Infrared Spectroscopy (FT-IR)

The coatings of LVA/SDS on the NWF were verified by FT-IR (Nicolet 6700, Thermo Scientific, Madison, WI, USA). SDS was dispersed in a KBr disk using the transmission mode scanned from 400 to 4000 cm^−1^ with a resolution of 4 cm^−1^. The FT-IR spectra of the NWF with or without LVA/SDS were recorded using attenuated total reflectance (ATR) mode from 600 to 4000 cm^−1^ with a resolution of 4 cm^−1^.

### 2.4. Oxygen Transmission Rate (OTR) of the NWF Coated with LVA/SDS

The effect of the coatings on oxygen permeability of the NWF was determined by a gas transmission rate test system (C230H, Labthink, Jinan, China) using a differential pressure method according to ASTM D3985-05, “Standard Test Method for Oxygen Gas Transmission Rate Through Plastic Film and Sheeting Using a Coulometric Sensor”, at 21 °C and 65% relative humidity [22]. The NWF (Yishun, Langfang, China) used in this test was 22 g/m^2^ and cut into round coupons with a diameter of 11 cm. The coupons were then coated with LVA/SDS as described above and tested eight times for 60 min each time. The OTR was calculated using the following equation [22], $\mathrm{OTR}=\frac{\left(E_{e}-E_{o}\right)\times Q}{A\times R_{L}}$, where $E_{e}$ is steady-state voltage level (mV), $E_{o}$ is zero voltage level (mV), *A* is specimen area (m^2^), *Q* is calibration constant (cm^3^·Ω/(mV·24 h)), and $R_{L}$ is the value of load resistance (Ω).

### 2.5. Determination of the Antibacterial Efficacy of NWF Coated with LVA/SDS

Two bacterial strains, including *Staphylococcus aureus* ATCC 6538, representing Gram-positive bacteria, and *Escherichia coli* ATCC 25922, representing Gram-negative bacteria, were used in this study. Both strains were stored at −20 °C in vials with tryptic soy broth (Hopebio, Qingdao, China) containing 25% glycerol (BBI Life Sciences, Shanghai, China). Before use, the bacteria were subcultured for three generations with incubation at 37 °C for 18 h, and a “sandwich test” was used to test the bactericidal effectiveness of the coated coupons [23]. An aliquot of 10 μL of the two bacterial suspensions (8.0–9.0 log CFU/mL) was individually placed in the center of the control or LVA/SDS coated square coupon, and a second identical coupon was placed on top. A sterile weight (100 g) was then placed on top of both coupons to ensure good contact between the two coupons. After different exposure times (3, 5, and 10 min), each pair of bacteria-bearing coupons was then individually placed in a 50-mL centrifuge tube containing 10 mL of 0.9% sterile saline and 30 glass beads (3-mm diameter). For the control coupons, the antimicrobial efficacy test with only 10 min of the contact time was carried out before placing them into the 50-mL tubes. The tubes were agitated by a Vortex mixer (HY-2, Rex, Shanghai, China) for 2 min to dissociate the cells from the NWF. One milliliter of the suspension and 0.1 mL of serial dilutions (1:10 in 0.9% sterile saline solutions) were plated in duplicate on tryptic soy agar (TSA, Hopebio) plates. The plates were incubated at 37 °C for 24 h, and typical colonies were counted. The experiment was performed twice with triplicate coupons each time.

### 2.6. Scanning Electron Microscopy (SEM)

The antibacterial efficacy of NWF treated with LVA/SDS was visualized using scanning electron microscopy (SEM). After being challenged with suspensions containing *S. aureus* and *E. coli* for a 10-min exposure time, as described previously, the coupons were then fixed with 2% glutaraldehyde (Macklin) for 4 h at 4 °C, and subsequently dehydrated in an ethanol series of 30, 50, 70, 90, and 100% for 10 min each. The coupons were sputtered with platinum (108 auto sputter coater, Ted Pella, Redding, CA, USA) and examined with a scanning electron microscope (Quanta 25, FEI, Hillsboro, OR, USA).

### 2.7. S. aureus Bioaerosol Challenge Test

**Figure 2:**
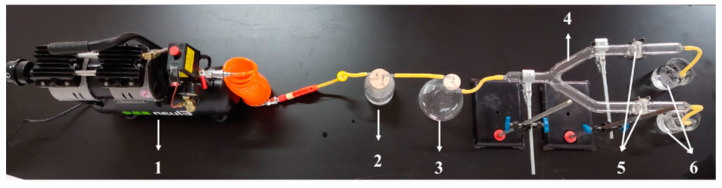
Structure of the bioaerosol dispenser and antibacterial testing of NWF. The device consists of the following parts: (**1**) air compressor; (**2**) bacterial suspension; (**3**) a filter flask for moisture insulation; (**4**) a Y-shaped chamber; (**5**) control and LVA/SDS-coated coupons, respectively; (**6**) bleach.

The LVA/SDS coated NWF was challenged with the bioaerosol of *S. aureus* based on ASTM F2101-19, “Standard Test Method for Evaluating the Bacterial Filtration Efficiency (BFE) of Medical Face Mask Materials, using a Biological Aerosol of *Staphylococcus aureus*” [24]. The apparatus for the bioaerosol dispenser was configured based on Demir, Cerkez, Worley, Broughton and Huang [15] with minor modifications as illustrated in Figure 2. After incubation at 37 °C for 18 h, 3 mL of *S. aureus* culture was suspended evenly in 300 mL of a 0.9% sterile saline solution (7.1 ± 0.1 log CFU/mL), in which an air compressor-connected nebulizer with a 0.22-µm filter in between was submerged for aerosol production. The NWF coated with or without LVA/SDS was cut into round coupons with a diameter of 3.70 cm (approximately 10.75 cm^2^) and respectively clamped into the parallel chambers of the Y-shape connecter in which the aerosolized bacteria were passing through. The air pressure was adjusted to 0.7–0.9 MPa through a pressure regulator mounted on the air compressor (E8L-550W, Eluan, Taizhou, China). The aerosolized bacteria were allowed to pass through the LVA/SDS-coated and control coupons for 6 h, and an average of 10 mL of the bacterial suspension was aerosolized. After a 6-h challenge, the air compressor was turned off and the system was set at a standstill for another 30 min. The coupons were then removed from the chambers and cell numbers were determined by spread plating serial dilutions (1:10 in 0.9% sterile saline solutions) onto plates as described above. The plates were incubated at 37 °C for 24 h before bacterial enumeration. The experiment was repeated three times.

### 2.8. Shelf-Life Stability Test

Coupons incorporated with 2% LVA + 1% SDS were used in this experiment. After being coated, as described above, the square NWF coupons were stored in Petri dishes on the laboratory bench at room temperature with ambient diurnal fluctuation. The stability of the coatings on NWF was determined by the aforementioned antibacterial efficacy test with 10 min of exposure time. The experiment was carried out at intervals for a total of 12 months with each time using four specimens.

### 2.9. Antiviral Efficacy Evaluation

The antiviral efficacy of 1% LVA + 0.5% SDS coated NWF was determined based on ISO 18184:2019 “Textiles—Determination of Antiviral Activity of Textile Products” [25] using the TCID_50_ method, which was chosen to check the dilution of the virus suspension that induces a cytopathic effect (CPE) in 50% of the cell unit. Madin-Darby canine kidney (MDCK) cell ATCC CCL-34 and influenza A virus subtype H1N1 (strain A/PR/8/34) were kindly provided by FeiFan standard technical service Co., Ltd., Suzhou, China. MDCK cells were cultured in 20 mL of growth medium (per 1 L of growth medium contains Kanamycin sulfate 60 mg, Eagle’s minimum essential medium (EMEM) 9.53 g, 7.5% sodium bicarbonate 15 mL, and inactivated fetal bovine serum 100 mL) in a flask, and then 0.1 mL of the cell suspension was deposited in each well of 96 well plates (Corning, Corning, NY, USA), which was subsequently placed in a CO_2_ incubator at a temperature of 37 °C for an incubation of 3 days before use. After the coating procedures, as described previously, LVA/SDS-coated and control NWF coupons (ca. 4 × 4 cm) were challenged with 0.2 mL of the H1N1 suspension (6.49 ± 0.03 log TCID_50_/mL) for a contact time of 2 h at 25 °C. The coupons were individually transferred to Stomacher bags (Jingxin, Shanghai, China) with nine volumes of maintenance medium (per 1 L of maintenance medium contains Kanamycin sulfate 60 mg, EMEM 9.53 g, and 7.5% sodium bicarbonate 15 mL). The bags were subsequently pummeled for 2 min at 240 rpm (Stomacher model LC-08, Jingxin). An aliquot of 0.1 mL of the supernatant was inoculated in the first eight wells, and 1:10 diluted suspensions were inoculated in the following wells with each dilution per eight wells. The final column with eight wells was inoculated with maintenance medium only as a negative control. The well plate was then incubated at 34 °C in the CO_2_ incubator for 1 h to let the cells absorb the virus. The supernatant was then drained, and the cells were washed with 0.1 mL of the maintenance medium. An aliquot of 0.2 mL of maintenance medium with Trypsin was added in each well and the plates were incubated at 34 °C for 7 days. CPE was observed and TCID_50_ was calculated by Behrens and Karber method [26]. The experiment was carried out in triplicates.

### 2.10. Statistical Analysis

The population of bacteria was converted to log CFU. Mean values were analyzed by one-way analysis of variance (ANOVA, version 25.0, SPSS Inc., Chicago, IL, USA) to identify significant differences (*p* < 0.05) with the Tukey test.

## 3. Results

### 3.1. Characterization and Air Permeability of NWF + LVA/SDS

**Figure 3:**
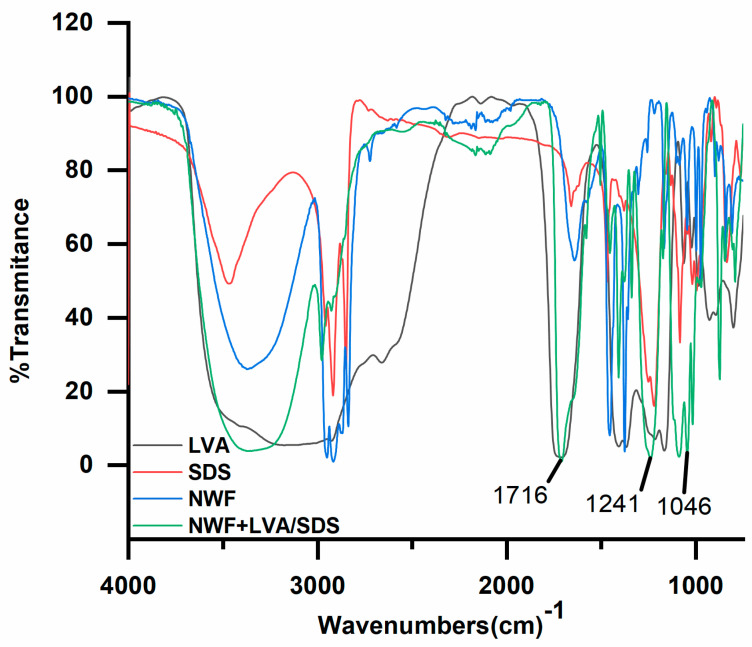
Fourier transform infrared spectroscopy (FT-IR) chromatograms of LVA, SDS, nonwoven fabrics (NWF), and LVA/SDS coated NWF (NWF + LVA/SDS).

The coating efficiency of LVA and SDS into the NWF was confirmed by FT-IR characterization (Figure 3). The prominent peaks of LVA and SDS compounds in the FT-IR spectrum were at 1716 (C=O), and 1241 (S=O) and 1046 (O-SO_3_) cm^−1^, respectively. Compared to the spectra of NWF, the presence of these specific peaks of LVA and SDS in the LVA/SDS treated samples indicated that LVA and SDS were successfully coated on the NWF.

As indicated in Table 1, the OTR of the control NWF was 469,417.7530 ± 25.6605 cm^3^/(m^2^ 24 h), which was not adversely affected by the coatings with the concentration of 0.5% LVA + 0.1% SDS (469,588.2685 ± 49.9348 cm^3^/(m^2^·24 h)) and 1% LVA + 0.5% SDS (469,642.9535 ± 41.2849 cm^3^/(m^2^·24 h)), but the OTR was remarkably reduced by the coating with 2% LVA + 1% SDS (698.4118 ± 26.6800 cm^3^/(m^2^ 24 h)).

### 3.2. Antibacterial Efficacy of LVA/SDS Coated NWF

**Figure 4:**
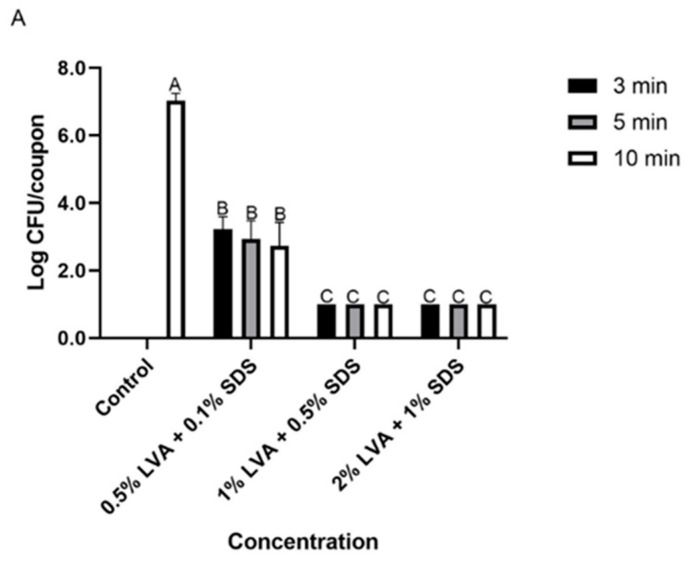

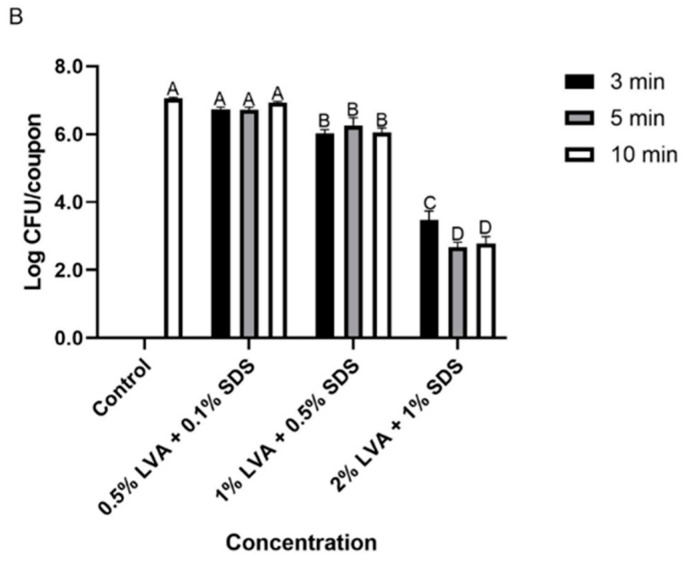
Antibacterial efficacy of NWF incorporated with different concentrations of LVA and SDS at different exposure times at 21 °C against *S. aureus* (**A**) and *E. coli* (**B**). The detection limit of the direct plating method was 1.0 log CFU/coupon. The bars represent the mean values + standard deviations (*n* = 6). Different letters above the bars indicate a significant difference (*p* < 0.05).

The antibacterial activity of LVA/SDS coated NWF was determined using the sandwich contact test. Generally, more cells were inactivated as the exposure times extended. For *S. aureus*, LVA/SDS-coated NWF showed superior antibacterial efficacy (Figure 4A). The bacterium recovered from the uncoated coupons was 7.0 ± 0.2 log CFU/coupon, while the fabrics loaded with 0.5% LVA + 0.1% SDS achieved >3.8-log CFU/coupon reduction at all the exposure times, and the bacterial counts were not recovered (<1.0 log CFU/coupon of the detection limit, > 6.0-log reduction) after 3-min contact time in the samples coated with higher concentrations (1% LVA + 0.5% SDS and 2% LVA + 1% SDS) of the combination. For *E. coli*, the bacterial population on the control coupons was determined as 7.1 ± 0.0 log CFU/coupon, and the cell counts (6.7–6.9 log CFU/coupon) of fabrics incorporated with low concentrations of the combination were not changed significantly (*p* > 0.05) after the contact times (Figure 4B). However, the coupons coated with medium and high concentrations of LVA and SDS achieved 1.1 and 3.6-log CFU/coupon reduction, respectively, after a treatment of 3 min. More than 4.3 log CFU of *E. coli* cells per coupon were decontaminated on 2% LVA + 1% SDS treated fabrics after a contact time of 5 and 10 min.

### 3.3. Microscopic Examination of LVA/SDS-Coated NWF

**Figure 5:**
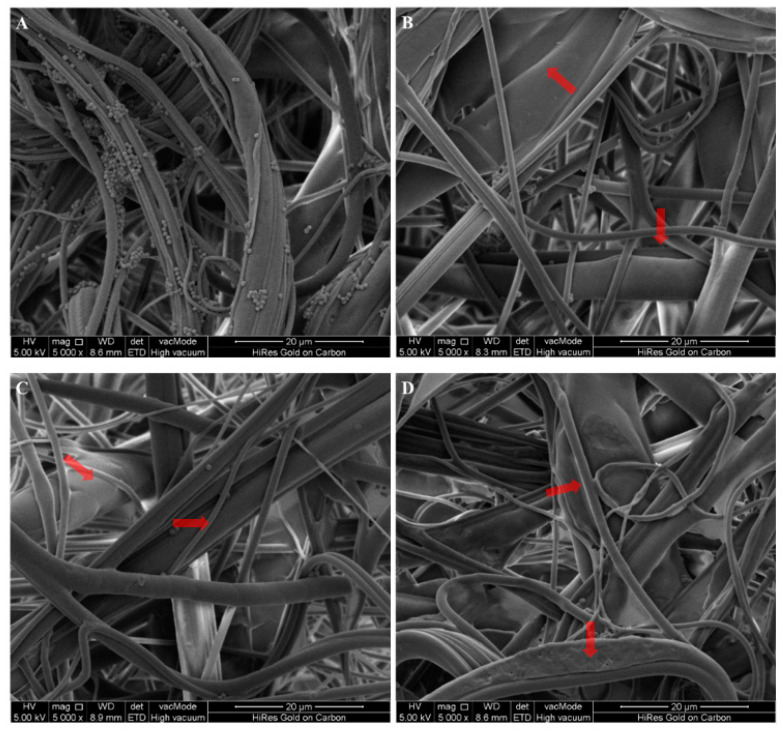
Representative photomicrographs by SEM of *S. aureus* after a 10-min exposure time on control coupons (**A**), and the coupons coated with 0.5% LVA + 0.1% SDS (**B**), 1% LVA + 0.5% SDS (**C**), and 2% LVA + 1% SDS (**D**). The red arrows highlight areas where cells detached after the 10-min contact of NWF + LVA/SDS.

**Figure 6:**
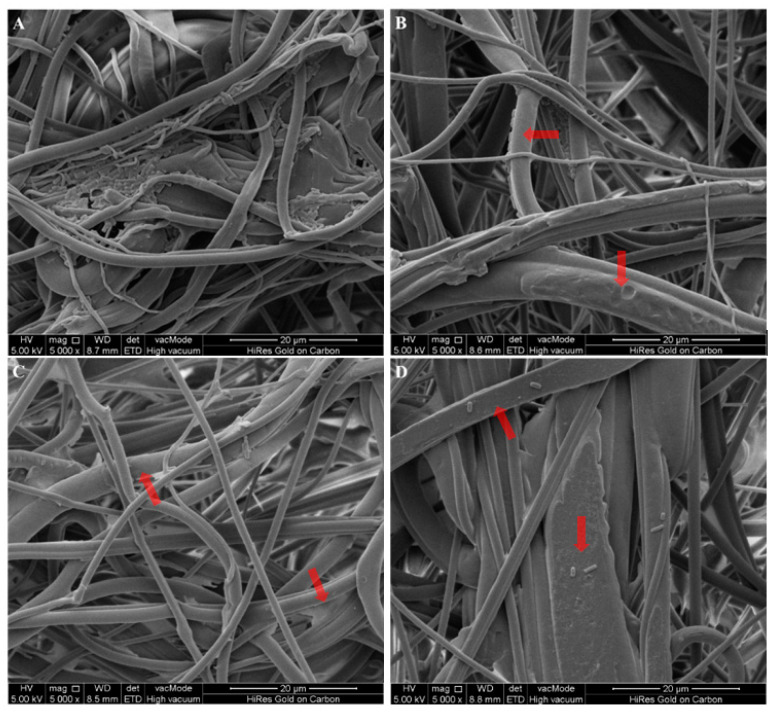
Representative photomicrographs by SEM of *E. coli* after a 10-min exposure time on control coupons (**A**), and the coupons coated with 0.5% LVA + 0.1% SDS (**B**), 1% LVA + 0.5% SDS (**C**), and 2% LVA + 1% SDS (**D**). The red arrows highlight areas where cells detached after the 10-min contact of NWF + LVA/SDS.

The antimicrobial efficacy of LVA/SDS-coated NWF against *S. aureus* and *E. coli* was also determined by SEM (Figure 5 and Figure 6). Microscopic images indicated that most of the cells in the meltdown polypropylene fabric matrix were detached after a 10-min contact with LVA/SDS-coated NWF. A greater number of both bacteria were removed when the treatment concentrations of LVA/SDS were increased. These images are consistent with the antibacterial assay and confirmed that the combination of LVA and SDS imparted antimicrobial properties to the NWF.

### 3.4. Antibacterial Efficacy of LVA/SDS Coated NWF against S. aureus Aerosols

In order to further evaluate the antibacterial effectiveness of the LVA/SDS-treated NWF, the coated and uncoated fabric coupons were challenged with *S. aureus* aerosols (Table 2). After 6-h residence time, the staphylococcal population of the control was 4.6 ± 0.1 log CFU/coupon, whereas the bacterial counts of the coated coupon, no matter whether treated with low or high concentrations of LVA and SDS, tested negative by the direct plating method with a detection limit of 1.0 log CFU/coupon. Results reveal that the LVA/SDS-coated fabric was able to effectively inactivate aerosol-borne bacteria attached to the NWF.

### 3.5. Shelf-Life Stability

The shelf-life stability of LVA/SDS-coated NWF was determined by testing its antibacterial effectiveness during 12 months of storage at room temperature. As indicated in Table 3, the LVA/SDS-coated NWF showed no significant (*p* > 0.05) loss of antibacterial efficacy against both *S. aureus* and *E. coli* during the whole storage period. Thus, the LVA/SDS-coated NWF showed superior shelf-life stability.

### 3.6. Antiviral Test

**Figure 7:**
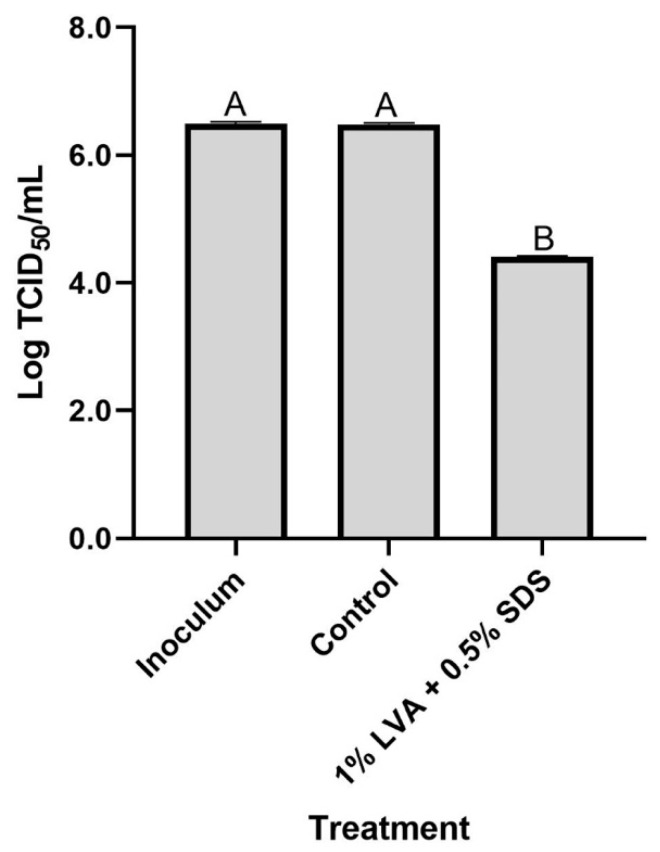
Efficacy of NWF coated with 1% LVA + 0.5% SDS of inactivating H1N1 virus. The NWF coupons treated with 75% ethanol only were the control. The bars represent the mean values + standard deviations (*n* = 3). Different letters above the bars indicate a significant difference (*p* < 0.05).

The antiviral activity of LVA/SDS-coated NWF was evaluated using the TCID_50_ method. As illustrated in Figure 7, virus recovered from the control coupons amounted to 6.48 ± 0.03 log TCID_50_/mL, with no significant (*p* > 0.05) difference from the inoculum (6.49 ± 0.03 log TCID_50_/mL). In contrast, fabrics coated with the binary combination exhibited a substantial reduction of 2.08 log TCID_50_/mL in the H1N1 virus, rendering >99% inactivation. These findings reveal the LVA/SDS-coated NWF is viricidal against the respiratory viral agent.

## 4. Discussion

The food industry is generally characterized as business where individuals work in close proximity to one another with operating environments being conducive to pathogen transmission between employees [2]. Thus, face masks and air filters are critical for not only protecting food workers but also preserving food safety, especially during the unprecedented COVID-19 pandemic caused by SARS-CoV-2. However, currently, commercial masks and filters used in the industry are made of plain fabrics without any biofunctions, and most of the ongoing studied antimicrobial fabrics are hardly feasible in the food industry due to cost-ineffectiveness, complex manufacturing, toxicity, and unstable performance [27]. To address these challenges, this study reports the development of fabrics with antimicrobial properties imparted by a coating of LVA and SDS, two compounds that are widely used in the food industry.

This study was designed to simulate a worst-case scenario to determine the antibacterial efficacy of the LVA/SDS coated fabrics by depositing bacterial inocula of *S. aureus* and *E. coli*, respectively, representing Gram-positive and Gram-negative bacteria, with an initial concentration as high as 7.0 log CFU/coupon. The different antimicrobial effectiveness of the LVA/SDS-coated fabrics against the two bacteria indicate that the coating is more bactericidal on Gram-positive bacteria than Gram-negative bacteria, the latter being more resistant to antimicrobials due to the presence of the additional protection afforded by the outer membrane [28]. The antibacterial mechanism of the combination is still not clear, but SDS can damage cell membranes by denaturing proteins, which may promote LVA pouring into cells causing a reduction of cytoplasmic pH by ionization of undissociated acid molecules [29,30].Several studies have been carried out to assess the use of inexpensive materials to coat fabrics to inactivate microorganisms. Affordable hand soap has been incorporated with fabrics, achieving an approximately 1.7-log TCID_50_/mL reduction of SARS-CoV-2 after a contact time of 1 min [27]. However, its antibacterial efficacy was not evaluated. Benzalkonium chloride, a sanitizer widely used in the food industry, has been coated on fabrics and was capable of inactivating >99% of SARS-CoV-2 particles in 1 min of contact [31]. In addition, a nonwoven face mask filter fabricated with a biofunctional coating of benzalkonium chloride was also challenged with Gram-positive bacteria, including methicillin-resistant *S. aureus* and *S. epidermidis*, by the agar disk diffusion test [31]. The agar disk diffusion method is a more qualitative other than quantitative method, and Gram-negative bacteria were not tested on the coated fabrics. Other low-cost materials, including NaCl, KCl, and K_2_SO_4_, have been coated on fabrics, and showed no greater antimicrobial activity (ca. 4-log CFU reduction) than LVA/SDS against bioaerosols [15,32]. N-halamine-coated fabrics have exhibited a substantial inactivation of bacteria and viruses, but the shelf-life of N-halamine-coated fabrics was less than 2 weeks due to a rapid chlorine loss when the fabrics were exposed to fluorescent light [15]. This drawback would adversely impact its application in the food processing environment on a large scale. Metals such as silver, copper, and zinc, have been incorporated into fabrics to restrict the spread of microbes [33]. Silver has been widely used for antimicrobial applications since ancient times [34]. Face masks presoaked for 5 to 7 h in colloidal silver solutions followed by drying were observed to render potent antimicrobial efficacy against *E. coli* and *S. aureus* with inhibition zones of >650 and 1000 mm^2^, respectively, for 50 and 100 mg/L of silver [35]. However, the presence of broad peaks in ultraviolet-visible absorption spectra for the colloidal silver and lumps in the SEM microphotographs revealed an uneven distribution and aggregation of the metal [35]. Recently, a new composite composed of a polymer matrix constructed from SiO_2_ anchored with silver nanoparticles exhibited remarkably antimicrobial efficacy against *E. coli*, *S. aureus*, as well as SARS-CoV-2, and has been proposed for use as a component for fabrication of face masks [36]. In another study, nonwoven fabrics permeated with copper oxide inactivated 99.9% of H1N1 and avian influenza virus (H9N2) after a 30-min contact [37]. More than 75% of SARS-CoV-2 was reduced by the presence of copper and copper oxide after 1 h of contact [11]. Compared to individual usage of metal or metal oxide, a combination of antimicrobial agents incorporated in fabrics was demonstrated to inactivate bacteria and viruses rapidly. The face mask comprised four layers with the first and fourth layers of spunbonded polypropylene (PP), the second sheet of cellulose/polyester infused with copper and zinc ions, and the third layer of meltblown PP. The first layer was treated with citric acid to create a low pH hydrophilic coating, which can induce structural rearrangements in the lipid layer of viruses. The second layer of cellulose can bind the virus through sulfhydryl and carboxyl groups [38]. The mask achieved more than 88% reduction for bacteria and 99.4% reduction of the viruses tested [38].

As evidenced by the above, much progress has been made toward the development of antimicrobial fabrics. However, most studies have focused on the decontamination of face masks from the clinical perspective, especially during the current COVID-19 pandemic. Limited research has underscored the need for antimicrobial fabrics from the food industry standpoint. Thus, fabrics treated with LVA and SDS were investigated. Although LVA and SDS have been endowed with GRAS status, the risk in the case of inhalation of the two chemicals by the face mask user is still not clear. The main ingredient, LVA, is a well-known cigarette additive, which is conducive to enhancing the perceptions of smoothness and mildness while smoking [39]. Moreover, face masks are usually made of at least three layers. In our study, LVA and SDS were only coated on the middle layer between two outer layers, with an interior layer that may block some chemicals inhaled into the respiratory system of the mask users. However, more research is needed to ensure their safe applications.

## 5. Conclusions

LVA/SDS-coated fabrics showed a potent antibacterial efficacy against *S. aureus* and *E. coli* with higher concentration and longer exposure time achieving greater reductions. After being challenged with *S. aureus* aerosols, LVA/SDS-coated fabrics inactivated the attached cells and exhibited potent decontamination capability. The antimicrobial fabrics achieved >99% of viral inactivation. In addition, the shelf-life of LVA/SDS-coated fabrics is very steady within 12 months, and oxygen permeability was not affected by the coating with a low and medium concentration of LVA and SDS. Therefore, fabrics coated with LVA/SDS have the potential to be applied in food processing plants and retail establishments to prevent the spread and transmission of airborne pathogens.

## Figures and Tables

**Table 1:** The oxygen transmission rate (OTR) of NWF incorporated with different concentrations of LVA/SDS.

Treatment of NWF	Oxygen Transmission Rate (OTR) ^a^ (cm^3^/(m^2^ 24 h))
Control	469,417.7530 ± 25.6605A ^b^
0.5% LVA + 0.1% SDS	469,588.2685 ± 49.9348A
1% LVA + 0.5% SDS	469,642.9535 ± 41.2849A
2% LVA + 1% SDS	698.4118 ± 26.6800B

^a^ Values are means ± standard deviations (*n* = 8). ^b^ Values in the same column that are not followed by the same letter are significantly different (*p* < 0.05).

**Table 2:** Biocidal efficacies of the NWF incorporated with different concentrations of LVA and SDS against *S. aureus* bioaerosols.

Treatment of NWF	Counts of Viable *S. aureus* (log CFU/Coupon) ^a^
Control	4.6 ± 0.1A ^b^
0.5% LVA + 0.1% SDS	<1.0B
2% LVA + 1% SDS	<1.0B

^a^ Values are means ± standard deviations (*n* = 3). The detection limit of the direct plating method was 1.0 log CFU/coupon. ^b^ Values in the same column that are not followed by the same letter are significantly different (*p* < 0.05).

**Table 3:** Shelf-life stability of the NWF incorporated with 2% LVA + 1% SDS.

Time (Month)	Bacterial Counts (log CFU/Coupon) ^a^	
	*E. coli*	*S. aureus*
Initial	2.8 ± 0.2	<1.00
1	2.7 ± 0.2	<1.00
2	2.8 ± 1.2	<1.00
4	2.4 ± 1.0	1.2 ± 0.3
6	2.3 ± 0.9	<1.00
12	2.6 ± 0.6	<1.00

^a^ Stability of the coatings on NWF was determined by the antibacterial efficacy test with 10 min of exposure time. Values are means ± standard deviations (*n* = 4). The detection limit of the direct plating method was 1.0 log CFU/coupon. No significant difference (*p* > 0.05) of the bacterial counts throughout the storage was observed compared to the control.

## Data Availability

Data are contained within the article.
